# Breast cancer risk in women with neurofibromatosis type 1: a register-based cohort study from Denmark and Sweden

**DOI:** 10.1016/j.breast.2026.104717

**Published:** 2026-01-30

**Authors:** Giorgio Tettamanti, Annie Pedersen, Maria Feychting, Bianca Tesi, Cecilie Ejerskov, Mia Aagaard Doherty, Emma Tham, Anna Skarin Nordenvall, Line Kenborg, Ann Nordgren

**Affiliations:** a Department of Molecular Medicine and Surgery, Karolinska Institutet, Stockholm, Sweden; b Unit of Epidemiology, Institute of Environmental Medicine, Karolinska Institutet, Stockholm, Sweden; c Department of Clinical Genetics and Genomics, Sahlgrenska University Hospital, Gothenburg, Sweden; d Department of Laboratory Medicine, Institute of Biomedicine, Sahlgrenska Academy, University of Gothenburg, Gothenburg, Sweden; e Clinical Genetics and Genomics, Karolinska University Hospital, Solna, Sweden; f Center for Hematology and Regenerative Medicine, Department of Medicine Huddinge, Karolinska Institutet, Stockholm, Sweden; g Centre for Rare Diseases, Department of Paediatric and Adolescent Medicine, Aarhus University Hospital, 8200, Aarhus N, Denmark; h Childhood Cancer Research Group, Danish Cancer Institute, 2100, Copenhagen E, Denmark; i Department of Radiology, Karolinska University Hospital, Stockholm, Sweden

**Keywords:** Neurofibromatosis type 1, Breast cancer, Risk, Survival, Epidemiology, Registries

## Abstract

**Background:**

An increased risk of breast cancer has been reported in women with neurofibromatosis type 1 (NF1), especially at younger ages, and NF1-related breast cancer has been associated with poor survival. We performed a large population-based cohort study to estimate the age-related breast cancer risk and survival in Danish and Swedish women with NF1.

**Patients and methods:**

We used national registers to identify all women with a diagnosis of NF1 in Denmark and Sweden born between 1930 and 1990 (Denmark) or 1987 (Sweden). Age- and sex-matched comparisons were randomly selected from population registers. Cox proportional hazards models were used to study the association between NF1, breast cancer risk, and overall 5-year mortality after a breast cancer diagnosis.

**Results:**

We included 2164 women with NF1 and 71 586 comparisons. A two-fold increased risk of breast cancer was observed in women with NF1 (hazard ratio (HR) 1.94, 95% confidence interval (CI) 1.59–2.36). The strongest association was observed in women between 30 and 39 years of age (HR = 3.98, 95% CI 2.16–7.32). Five-year mortality after a breast cancer diagnosis was higher in women with NF1 (HR = 1.98, 95% CI 1.31–2.99).

**Conclusions:**

Our results suggest that although increased, the risk of breast cancer in women with NF1 is not as high as previously reported, particularly among young women. These findings add to previous data and will contribute to the gathered knowledge needed to accurately address the risk of breast cancer in women with NF1.

## Highlights

• Women with neurofibromatosis type 1 (NF1) have a two-fold increased risk of breast cancer at any age.
• The strongest association, 4-fold increased risk, was observed among women between 30 and 39 years of age.
• The cumulative incidence of breast cancer by age 40 among women with NF1 was low (1.3% in Sweden and 1.6% in Denmark).
• Women with NF1 have a two times increased 5-year mortality after a breast cancer diagnosis.

## 1 Introduction

Neurofibromatosis type 1 (NF1) is a genetic multisystem disorder characterized by dermatological, skeletal and cognitive manifestations as well as an increased risk of benign and malignant tumors. NF1 is caused by heterozygous pathogenic loss-of-function variants in, or a deletion of, the tumor-suppressor gene *NF1,* resulting in neurofibromin protein deficiency and increased signaling of the Ras/mitogen-activated protein kinase (MAPK) pathway. Upon somatic mutation of the second, wild-type, *NF1* allele there is risk of tumor development in accordance with Knudson's two-hit hypothesis [1]. Consequently, individuals with NF1 present with a generally increased risk of tumor development including malignant peripheral nerve sheath tumor, peripheral and central nervous system tumors, phaeochromocytomas, gastrointestinal stromal tumors and breast cancer, and current guidelines recommend long-term tumor surveillance[2, 3].

An increased risk of breast cancer in women with NF1 has been reported in several cohorts [[4], [5], [6], [7], [8], [9]]. Specifically, women at younger ages seem to be at increased risk [[4], [5], [6], [7], [8], [9], [10]]. In a population-based Finnish cohort including 737 women with NF1, a standardized incidence ratio (SIR) of nearly 3 was observed when examining breast cancer occurrence at any age while among women under 40 years of age a SIR greater than 14 was found [10]. Similarly, a meta-analysis including four studies and a total of 4178 women with NF1 reported a SIR of 3 when comparing women with NF1 to the general population, but a SIR of 5 was observed in women younger than 50 years of age [11]. Among women with NF1 over 50 years of age, findings across cohorts are inconsistent, with some studies showing an elevated risk [4, 9] and others reporting risks similar to the general population [5, 8]. Moreover, breast cancer in NF1 has been associated with a worse prognosis and shorter survival compared to non-NF1 breast cancer [10, 12]. Given the reported increased risk of breast cancer at young ages, international guidelines recommend yearly breast magnetic resonance imaging (MRI) or mammogram screening from the age of 30–50 years, thereafter screening in accordance to national guidelines [2, 3]. However, with few exceptions, the reported data on NF1 and breast cancer has been based on small numbers of cases and only few studies are population-based. Thus, the magnitude of risk is not well established and the evidence underlying current recommendations is moderate [2, 3]. Given the large psychological consequences of living with a cancer predisposition syndrome and adhering to surveillance programs, there is a need to more accurately assess the breast cancer risk in women with NFI, including the risk at different ages. To this end, additional large-scale population-based data are needed to improve counselling and to design adequate surveillance programs.

Here, we gathered data from Denmark and Sweden to perform a large population-based matched cohort study with more than 2000 women with NF1 to estimate the breast cancer risk at specific ages and determine if women with NF1 face higher mortality after a breast cancer diagnosis compared to other women with breast cancer.

## 2 Methods

### 2.1 Identification of women with NF1

The association between breast cancer risk and NF1 was studied using a matched cohort design with data from Danish and Swedish national registers. We identified all women in Denmark and Sweden born between 1930 and 1987 (1990 in Denmark) who had a diagnosis of NF1. In Sweden, women with NF1 were identified using information from the National Patient Register, a register that contains information on inpatient care since 1964 and achieved nationwide coverage of inpatient care diagnoses in 1987; moreover, this register also has nationwide data on outpatient care diagnoses since 2001 [13]. In addition to the National Patient Register, for the Stockholm region, which corresponds to approximately 25% of the Swedish population, we additionally identified women with genetically confirmed diagnosis of NF1 using data from the Laboratory Information Systems (LIS) at the Karolinska University Hospital. We also used this source of data to exclude women who had a neurofibromatosis diagnosis in the National Patient Register but had a diagnosis of neurofibromatosis type 2, now called more correctly NF2-related schwannomatosis (from here on referred to as NF2), in LIS: only 5 individuals with a neurofibromatosis diagnosis in the Swedish National Patient Register have an NF2 diagnosis in LIS. In Denmark, hospital admissions from the Danish National Patient Register (established in 1977) mentioning neurofibromatosis as a discharge diagnosis and the NF1 Registry cohort were used to identify women with NF1 [14]. In the International Classification of Diseases (ICD) used in the Danish and Swedish registers it is not possible to distinguish between the diagnoses NF1 and NF2. For this reason, women with neurofibromatosis according to the Danish and Swedish patient registers were classified as NF1 if there was not a concomitant diagnosis of a tumor that is more common among individuals with NF2, such as meningiomas, vestibular schwannomas, or other cranial nerve tumors (excluding optic nerve tumors) [15].

### 2.2 Creation of matched cohort study

After having identified Swedish women with a diagnosis of NF1, we used data from the Swedish Total Population Register [16] to randomly select fifty matched comparisons for each woman with NF1 using sex and birth year as matching factors. In Denmark, ten comparisons were randomly selected using the Danish Civil Registration System and matched to NF1 women using sex and birth month and year. In both countries, the matched comparisons were alive and living in Denmark/Sweden at index date (i.e., first date of NF1 diagnosis). The Danish and Swedish matched cohorts were then linked to the respective national cancer registers [17, 18] to obtain information on cancer diagnoses for all women included in the study: this information was available until the end of 2017 for Sweden and end of 2022 for Denmark. Only malignant breast cancer diagnoses were considered in this study.

### 2.3 Statistical analysis

When studying breast cancer risk, all women were followed from index date, or their 20th birthday if their index date was before this date, until first breast cancer diagnosis, emigration, death, or end of the study period (end of 2017 for Sweden and end of 2022 for Denmark), whichever occurred first. Dates of emigration and death were available until the end of 2019 for Sweden and end of 2022 for Denmark and were obtained from the population registers. We also obtained information regarding main cause of death from the causes of death registers [19, 20]. Women with a breast cancer diagnosis before index date were removed from the main analysis. Stratified Cox proportional hazards models using attained age as the underlying time scale were used to study the association between NF1 and breast cancer risk. The analyses on breast cancer risk were inherently adjusted for the matching factors. We first assessed breast cancer risk from age 20 onwards and then at specific ages (20–39, 30–39, 40–49, 50–59, 60+). In a sensitivity analysis, we considered all first breast cancer diagnoses including the ones occurring before the index date. In an additional analysis, we used data from the Swedish Total Population Register to adjust for age at first childbirth (no children, age at first childbirth <30, age at first childbirth ≥30) and parity (no children, 1 child, 2 or more children): due to data availability, this analysis was performed only on the Swedish data. We also performed a secondary analysis including only women who were born from 1950, to determine whether similar associations between NF1 and breast cancer risk were observed when restricting the analyses to more recent birth cohorts.

A Cox proportional hazards model using time since diagnosis as the underlying time scale was used to study survival after a breast cancer diagnosis (5-year overall survival). Analyses on 5-year overall survival were adjusted for age at breast cancer diagnosis (<40, 40–49, 50–59, 60+) and decade of diagnosis (<1980, 1980–1989, 1990–1999, 2000–2009, 2010-). We first performed an analysis including all breast cancer cases and then we stratified by age at breast cancer diagnosis (<50/50+).

Danish and Swedish data were analyzed separately and pooled using a random-effect meta-analysis. Statistical heterogeneity was evaluated with the Cochran Q-test and the I-squared statistic [21]. All results are reported as hazard ratios (HRs) with their corresponding 95% confidence intervals (CIs). Cumulative incidences at specific ages were calculated using the Aalen-Johansen estimator, considering death as a competing event and using age as the underlying time scale. Data management was performed using SAS 9.4, while Stata 15.1 was used for all statistical analyses. Ethical approval was granted from the Stockholm Regional Ethics Committee (dnr 2015/608–31/4), and the study is registered in the Danish Cancer Institute's internal project database (journal number 2019-DCRC-0063) in agreement with the General Data Protection Regulation.

## 3 Results

Our study includes 2164 women with NF1 (888 in Denmark and 1276 in Sweden) and 71 586 comparisons. Descriptive characteristics of the women included in this study are reported in Table 1. In total we identified 113 malignant breast cancer cases among women with NF1 and 2051 in the matched comparison group. In Sweden, 4.1% of the NF1 women had a breast cancer diagnosis after index date, compared to 2.7% of the matched comparisons; a higher proportion of breast cancer cases was observed in the Danish data (6.9% in the NF1 group and 4.4% among the comparisons) despite a rather similar birth year distribution in the two countries. Women with NF1 had a lower median age at breast cancer diagnosis (53 years in Sweden and 50 in Denmark) compared to the matched comparisons (59 years in both countries). The youngest age at breast cancer diagnosis was 31 years among women with NF1 and 24 years among the comparisons. The total number of person-years and number of breast cancer cases observed among the women included in this study is reported in Table 2.

**Table 1:** Descriptive characteristics of women with neurofibromatosis type 1 (NF1) and matched comparisons.

	Matched comparisons N (%)	NF1 N (%)
**Sweden**		

	62 889	1276

**Birth year**		
1930–1939	7266 (11.6)	149 (11.7)
1940–1949	9944 (15.8)	204 (16.0)
1950–1959	10 270 (16.3)	208 (16.3)
1960–1969	12 361 (19.7)	249 (19.5)
1970–1979	13 239 (21.1)	267 (20.9)
1980–1987	9809 (15.6)	199 (15.6)

N breast cancer diagnosis (%)	1665 (2.7)	52 (4.1)
Median age at breast cancer diagnosis	59	53
Average age at breast cancer diagnosis (SD)	58.5 (11.5)	52.8 (10.5)

**Denmark**		

	8697	888

**Birth year**		
1930–1939	847 (9.7)	87 (9.8)
1940–1949	1302 (15.0)	133 (15.0)
1950–1959	1418 (16.3)	144 (16.2)
1960–1969	1783 (20.5)	180 (20.3)
1970–1979	1737 (20.0)	178 (20.0)
1980–1990	1610 (18.5)	166 (18.7)

N breast cancer diagnosis (%)	386 (4.4)	61 (6.9)
Median age at breast cancer diagnosis	59	50
Average age at breast cancer diagnosis (SD)	59.3 (11.6)	51.9 (11.5)

**Table 2:** Total number of person-years and number of breast cancer cases among women with neurofibromatosis type 1 (NF1) and their matched comparisons in Denmark and Sweden.

	NF1			No NF1		
	N women	Person-years	N breast cancer	N women	Person-years	N breast cancer
**Sweden**						

**Years**						
1970–1999	781	4803.9	14	40230	268996.5	284
2000–2009	906	6111.4	22	47776	345986.4	548
2010-	1083	7128.7	16	59 094	406700.6	833

**Age**						
20–39	725	7323.4	6	36071	376277.1	96
30–39	696	4121.4	6	35090	215326.9	84
40–49	656	3989.5	12	34218	219760.4	276
50–59	543	3244.7	18	29438	193862.5	488
60+	417	3490.5	16	24012	232014.1	805

**Denmark**						

**Years**						
1970–1999	440	3482.3	13	4332	39178.0	61
2000–2009	704	4922.6	17	7399	54197.5	111
2010-	730	8168.4	31	7840	92333.5	214

**Age**						
20–39	571	6685.9	7	5607	65249.5	21
30–39	543	3859.0	7	5306	38241.8	<21
40–49	551	3766.2	22	5642	41176.3	62
50–59	446	3007.0	18	5007	36895.3	119
60+	307	3116.9	14	3685	42416.8	184

### 3.1 NF1 and breast cancer risk

A two-fold increased risk of breast cancer at any age was observed among women with NF1 (HR = 1.94, 95% CI 1.59–2.36). When studying the risk of breast cancer at specific ages, a three times increased risk of breast cancer was observed in young adulthood (20–39 years of age) (HR = 3.38, 95% CI 1.86–6.17) (Table 3). No women with NF1 had a breast cancer diagnosis before 30 years of age, and when looking at the age group 30–39 a 4-fold increased risk of breast cancer was found in women with NF1 (HR = 3.98, 95% CI 2.16–7.32): however, the cumulative incidence of breast cancer before age 40 was only 1.3% in Sweden and 1.6% in Denmark, compared to 0.4% and 0.5% among the matched comparisons (Table 4). Women with NF1 had a three-fold and two-fold increased breast cancer risk at age 40–49 and 50–59 respectively, while no association was observed in women aged 60+ (Table 3).

**Table 3:** Association between neurofibromatosis type 1 (NF1) and breast cancer risk.

Age	N breast cancer cases NF1/no NF1	HR (95% CI)
20–39	13/127	3.38 (1.86–6.17)
30–39	13/<105∗	3.98 (2.16–7.32)
40–49	34/338	3.14 (2.07–4.77)
50–59	36/607	1.97 (1.39–2.78)
60+	30/989	1.20 (0.83–1.74)
Any age	113/2051	1.94 (1.59–2.36)

**Sweden**		

20–39	6/96	3.46 (1.51–7.94)
30–39	6/84	4.05 (1.76–9.33)
40–49	12/276	2.47 (1.38–4.41)
50–59	18/488	2.19 (1.37–3.52)
60+	16/805	1.33 (0.81–2.18)
Any age	52/1665	1.94 (1.47–2.56)

**Denmark**		

20–39	7/21	3.30 (1.38–7.86)
30–39	7/<21^a^	3.90 (1.60–9.51)
40–49	22/62	3.79 (2.30–6.23)
50–59	18/119	1.74 (1.05–2.88)
60+	14/184	1.05 (0.60–1.84)
Any age	61/386	1.94 (1.47–2.56)

Inherently adjusted for birth year.

a The actual number of cases in this group cannot be reported because of the Statistics Denmark's restrictions.

**Table 4:** Cumulative incidence of breast cancer at specific ages in Denmark and Sweden.

	NF1		No NF1	
	Breast cancer cases	Cumulative incidence (95% CI)	Breast cancer cases	Cumulative incidence (95% CI)
**Sweden**				

Until age 40	6	1.3% (0.5%–2.7%)	96	0.4% (0.4%–0.5%)
Until age 50	18	3.7% (2.3%–5.6%)	372	1.7% (1.5%–1.9%)
Until age 60	36	7.3% (5.2%–9.9%)	860	4.1% (3.8%–4.3%)
Until age 70	49	10.4% (7.9%–13.4%)	1350	7.1% (6.7%–7.5%)
Any age	52	11.3% (8.6%–14.4%)	1665	11.0% (10.4%–11.6%)

**Denmark**				

Until age 40	7	1.6% (0.7%–3.2%)	21	0.5% (0.4%–0.8%)
Until age 50	29	6.3% (4.3%–8.8%)	83	2.0% (1.6%–2.5%)
Until age 60	47	10.2% (7.7%–13.1%)	202	5.0% (4.4%–5.7%)
Until age 70	57	12.9% (9.9%–16.2%)	321	9.0% (8.1%–10.0%)
Any age	61	14.8% (11.4%–18.6%)	386	13.7% (12.1%–15.4%)

In analyses stratified by country, similar results were observed, with the strongest association observed in women aged 30–39 (HRs = 3.90 and 4.05, in Denmark and Sweden respectively) and no increased risk was observed among women aged 60+. In women aged 40–49, a stronger association was observed in Denmark compared to Sweden (HR = 3.79 and 2.47) (Table 2). In the Swedish data we could adjust for parity and age at first childbirth: similar results were observed after this additional adjustment (Supplementary Table 1). While in the main analysis the HR observed among women aged 50–59 was 2.19, after adjusting for parity and age at first childbirth the HR was only slightly attenuated (HR = 2.12, 95% CI 1.32–3.41).

In the sensitivity analysis in which all breast cancer diagnoses were included, even the ones occurring before the index date, the results were similar to what we observed in the main analysis (Supplementary Table 2). As in the main analysis, women with NF1 had a two-fold increased risk of breast cancer at any age (HR = 1.92, 95% CI 1.62–2.29) and the strongest association was observed in women aged 30–39. When restricting the analysis to women born from 1950 onwards, similar associations were observed for the age groups 20–39 and 40–49, while an attenuation was observed for the age group 50–59, where the HR decreased from 1.97 in the main analysis to 1.54 (Supplementary Table 3).

### 3.2 NF1 and 5-year survival after a breast cancer diagnosis

Within five years after cancer diagnosis, 25% of the women with breast cancer and NF1 had died, compared to 13% of women with breast cancer without NF1 (Table 5). Among women who died within five years after the breast cancer diagnosis, in the NF1 group 71% of them had cancer as the main cause of death, while this proportion was a bit higher among women without NF1 (81%). In adjusted analyses, we found a two-fold higher mortality among breast cancer patients with NF1 compared to breast cancer patients without NF1 (HR = 1.98, 95% CI 1.31–2.99); similar findings were observed when stratifying by age at breast cancer diagnosis (Table 5). When performing country-specific analyses, different results were observed in Sweden and Denmark. Among women diagnosed with breast cancer below age 50 years, while a two-fold increased mortality was found in Swedish breast cancer patients with NF1 (HR = 2.39, 95% CI 0.94–6.10), no association was found in Denmark (HR = 1.45, 95% CI 0.49–4.28) (Table 5). Instead, among women diagnosed with breast cancer after 50 years of age, only indications of an increased mortality were found in the Swedish data (HR = 1.65, 95% CI 0.77–3.53) while the association was stronger in Denmark (HR = 2.66, 95% CI 1.37–5.18). No statistically significant heterogeneity between Denmark and Sweden was found in any of the analyses.

**Table 5:** Results from Cox regression models on five-year mortality in women with and without neurofibromatosis type 1 (NF1) after a breast cancer diagnosis, overall and stratified by age at diagnosis.

	N breast cancer cases/N deaths	HR (95% CI)
Any age		
No NF1	2051/274	1 (ref)
NF1	113/28	1.98 (1.31–2.99)
Below age 50		
No NF1	455/58	1 (ref)
NF1	47/10	1.93 (0.95–3.92)
Above age 50		
No NF1	1596/216	1 (ref)
NF1	66/18	2.16 (1.31–3.57)

**Sweden**		

Any age		
No NF1	1665/217	1 (ref)
NF1	52/12	1.79 (1.00–3.22)
Below age 50		
No NF1	372/48	1 (ref)
NF1	18/5	2.39 (0.94–6.10)
Above age 50		
No NF1	1293/169	1 (ref)
NF1	34/7	1.65 (0.77–3.53)

**Denmark**		

Any age		
No NF1	386/57	1 (ref)
NF1	61/16	2.19 (1.23–3.91)
Below age 50		
No NF1	83/10	1 (ref)
NF1	29/5	1.45 (0.49–4.28)
Above age 50		
No NF1	303/47	1 (ref)
NF1	32/11	2.66 (1.37–5.18)

Adjusted for age at breast cancer diagnosis and decade of diagnosis.

## 4 Discussion

In this large population-based matched cohort study including more than 2000 women with NF1, we found that women with NF1 have a two-fold increased risk of breast cancer and a lower median age at breast cancer diagnosis compared to the general population. A stronger association was observed among young women, as a four times increased breast cancer risk was observed in women aged 30–39 years old. However, the cumulative incidence of breast cancer in young women with NF1 was low, only 1.3% and 1.6% in Sweden and Denmark respectively. No increased risk was observed in women over 60 years of age. Finally, women with NF1 had a two-fold increased mortality in the five-year period following a breast cancer diagnosis.

The findings of the present study are in line with previous reports showing increased risk of breast cancer in women with NF1, especially at younger ages [[4], [5], [6], [7], [8], [9]]. However, our results suggest that the magnitude of risk in women with NF1 below the age of 40 years might not be as high as previously thought. In a population-based Finnish cohort of 1404 individuals with NF1, whereof 737 women, a standardized incidence ratio (SIR) of 2.82 (95% CI 1.92–4.00) was reported for breast cancer at all ages (similar to our HR of 1.94). The highest SIR in the Finnish study was observed in women <40 years of age (SIR 14.25, 95% CI 6.51–27.04) and in women aged 30–39 years the reported breast cancer risk was 4.74% [10], while in our study the cumulative incidence by age 40 was below 2%. In a meta-analysis including four studies [[5], [6], [7], 9] and a total of 4178 women with NF1, a SIR of 3was reported in women with NF1 compared to the general population, while a SIR of 5 was observed among women younger than 50 years of age, which is more in line with our results, although slightly higher than what we have observed [11]. Based on these published breast cancer risk estimates, international guidelines recommend yearly breast magnetic resonance imaging (MRI) or mammogram screening from the age of 30 years in women with NF1 [2, 3, 22, 23]. While there are benefits of early screening, these need to be weighed against potential risks. Although radiation exposure is low with mammography, there is a risk of radiation-related secondary malignancies in NF1 [24], and the safety of early mammography screening in this population is not known. Breast MRI has a higher sensitivity, but lower specificity compared to mammography [25], leading to a potential risk of false positives, unnecessary biopsies, and negative psychological consequences. Studies investigating benefits and risks of early mammography and MRI screening in NF1 are needed as well as studies to improve individual risk prediction. There is data suggesting a genotype-phenotype correlation in relation to breast cancer risk in NF1 [26]. However, there is also a large intrafamilial phenotypic variation, and future studies exploring the genetic and environmental factors underlying intra- and interfamilial variation are warranted. Meanwhile, an individualized approach based on clinical factors could be considered. In Sweden, where population mammography screening is recommended from the age of 40, current national NF1 guidelines do not routinely recommend breast imaging for women between 30 and 40 years of age. Instead, self-breast examination and referral to a unit with experience in hereditary breast cancer is recommended for individualized recommendations considering factors such as family history, high mammographic density, and the occurrence of neurofibromas in the breast, which makes both self-examination and interpretation of mammography images difficult [27]. In Denmark, population mammography screening is recommended from the age of 50 years, but women with NF1 are routinely screened from 40 years of age. Regardless of differences in general national guidelines, individual attention and liberal investigation of any lumps or other suspicious signs detected by self-examination should be applied in women with NF1. In country-specific analysis we observed that among women aged 40–49 the HR was lower in Sweden (2.47) than in Denmark (3.79). Although these HRs are quite comparable, it is possible that the differences in mammography screenings in the two countries could explain, at least in part, the observed results.

In our study, the five-year survival after a breast cancer diagnosis was 75% for women with NF1 and 87% for comparisons. As compared to the present study, results from the population-based Finnish cohort including 737 women with NF1 showed a poorer five-year survival in NF1 women, 68%, but 82% in the general population [10]. In line with these data, a poorer breast cancer survival in NF1 compared to non-NF1 women has been reported in other European cohorts, with an association similar in magnitude to what we have observed in our study [12]. Possible explanations have been suggested and include associations to unfavorable prognostic factors in NF1 breast cancers such as oestrogen and progesterone negativity and HER2 amplification [10, 12]: information on tumor characteristics was not available in our study. Other factors contributing to the poor prognosis could be a delay in diagnosis due to a younger age at onset and neurofibromas in the breast that impair palpation and mammography interpretation. Moreover, individuals with NF1 have an increased mortality also from other causes and a shorter life expectancy than the general population [28, 29]. In our study we found that among women who died within five years from the breast cancer diagnosis, a lower proportion had cancer as the main cause of death in the NF1 group compared to other women with breast cancer (71% vs 81%). Finally, an unexpected finding in our data was the country-specific results on mortality in women under 50 years of age. In these analyses we observed a two-fold increased five-year mortality in Swedish NF1 women, but no significant difference in mortality between Danish women with NF1 and comparisons. One possible explanation for this difference is that, as mentioned in the previous paragraph, while in Sweden breast cancer screening in the general population starts at age 40, in Denmark it starts at age 50. Moreover, Danish women with NF1 are followed up at one of two national centers for rare diseases and are referred to breast cancer screening by their center physician from age 40. This means that young Danish women with NF1 are likely to receive an earlier breast cancer diagnosis, which might result in a better prognosis These results need to be further explored as they are based on small numbers (18 breast cancer cases with NF1 in Sweden, 5 deaths; 29 breast cancer cases with NF1 in Denmark, 5 deaths) and therefore cannot exclude chance as a possible explanation for this difference. Support for this possible explanation is provided by the study performed by Evans et al., in which they reported that a screened population of women with NF1 had a better breast cancer prognosis than the other NF1 cohorts that were not screened: however, the screened cohort included only 27 women with breast cancer and none of them died [12].

The main strengths of the present study are the large cohort size, the population-based design, and the usage of data from high quality nationwide health registers. An additional strength of this study is the consistency of results when including breast cancer diagnoses that occurred prior to the index date; these findings closely mirrored those from the main analyses. This suggests that limiting the analysis to cancer diagnoses after the index date did not lead to an underestimation of the association between NF1 and breast cancer risk.

One limitation of the current study is that, since the ICD version used in the two countries does not distinguish between NF1 and NF2, there is a risk that we might have classified some women with NF2 as NF1. However, NF2 is rare compared to NF1 and to minimize the risk of misclassification, we excluded individuals with NF2 related tumors such as meningiomas and cranial nerve tumors. However, since meningiomas are not rare in NF1, it is possible that we have erroneously removed few individuals with NF1 who had a diagnosis of meningioma. In Sweden, we also excluded individuals who had received a genetic diagnosis of NF2 at the Karolinska University Hospital: however, this data is available only for the Stockholm region which covers approximately 25% of the total population. In Denmark half of the women with NF1 were identified using the NF1 Registry, where only individuals with a confirmed NF1 diagnosis are included. Further, there is a risk that mild cases of NF1 have been undiagnosed and therefore not included in our cohort. Thus, the presented risks of breast cancer might not be generalizable to the whole population of women with NF1 and may represent an overestimation of the true association. Moreover, we cannot exclude the possibility that some women identified as having NF1 could have received an erroneous neurofibromatosis diagnosis which could have led to the inclusion of healthy women in the NF1 group (possibly leading to an underestimation of the association) or that women with other syndromes were wrongly diagnosed as having NF1, which could have biased the association. Another limitation of this study is that some of the women with NF1 born in the first decades of the study could have died before they had a chance to have a neurofibromatosis diagnosis reported to the patient registers. To minimize this selection bias, our main analyses used delayed entry at the date of first recorded NF1 diagnosis. Consequently, women contribute person-time only after NF1 is ascertained, and the age-stratified analyses are constructed so that the 20–39 age group includes only women whose NF1 was recorded before age 40, which means that this group mainly constitutes women born in more recent decades. This design intentionally avoids mixing older birth cohorts into the young-age stratum: we therefore believe that the impact of selection bias on our main analysis is likely limited. Since this was a register-based study, we did not have information on tumor characteristics or information regarding lifestyle factors that could have influenced breast cancer risk and survival, such as smoking and diet among others; we also did not adjust for achieved education despite the fact that individuals with NF1 have a lower educational level than the general population [30]. However, it is important to remark that lifestyle factors and educational level do not confound the associations between NF1 and breast cancer risk and survival since they are mediators, as individuals are born with NF1. In the Swedish data we had the opportunity to additional adjust for parity and age at first childbirth, two important risk factors for breast cancer occurrence. Only a small attenuation in the associations was observed after adjusting for these factors: this suggests that these mediating factors had little effect on the association between NF1 and breast cancer in women.

In conclusion, we found an increased breast cancer risk in women with NF1, but not as high as previously reported, especially among young women. Even though young women (age 30–39) with NF1 had a four-fold increased risk of breast cancer, the breast cancer cumulative incidence by age 40 was below 2%. In line with previous data, we also found an increased mortality five years after breast cancer diagnosis. Our findings add to previous data and will contribute to the gathered knowledge needed to accurately address the risk of breast cancer in NF1 in order to improve counselling and provide evidence-based guidelines for this specific population.

## CRediT authorship contribution statement

**Giorgio Tettamanti:** Writing – review & editing, Writing – original draft, Visualization, Project administration, Formal analysis, Data curation, Conceptualization. **Annie Pedersen:** Writing – review & editing, Writing – original draft, Visualization, Conceptualization. **Maria Feychting:** Writing – review & editing, Methodology. **Bianca Tesi:** Writing – review & editing. **Cecilie Ejerskov:** Writing – review & editing. **Mia Aagaard Doherty:** Writing – review & editing. **Emma Tham:** Writing – review & editing, Visualization. **Anna Skarin Nordenvall:** Writing – review & editing, Data curation. **Line Kenborg:** Writing – review & editing, Visualization, Project administration, Methodology, Funding acquisition, Formal analysis, Data curation. **Ann Nordgren:** Writing – review & editing, Writing – original draft, Visualization, Supervision, Project administration, Funding acquisition, Data curation, Conceptualization.

## Consent to participate declaration

In Sweden and Denmark informed consent from the individuals included in register-based studies is not needed.

## Funding

This study was supported by the 10.13039/501100004359Swedish Research Council, the 10.13039/501100002794Swedish Cancer Society, and 10.13039/501100009708Novo Nordisk Foundation.

## Declaration of competing interest

The authors declare that they have no known competing financial interests or personal relationships that could have appeared to influence the work reported in this paper.
