# Supplementary material for: Breast cancer risk in women with neurofibromatosis type 1: a register-based cohort study from Denmark and Sweden
Source: Breast. 2026 Jan 30;86:104717. doi: 10.1016/j.breast.2026.104717 (PMC12905740; doi:10.1016/j.breast.2026.104717)
Supplement: Multimedia component 1 [file mmc1.docx]

Supplementary Table 1: Breast cancer risk in women with neurofibromatosis type 1 (NF1) after adjusting for parity and age at childbirth

| **Age** | **N breast cancer cases**  **NF1/no NF1** |  | **HR (95% CI)** |
| --- | --- | --- | --- |
| 20-39 | 6/96 |  | 3.16 (1.36-7.32) |
| 30-39 | 6/84 |  | 3.87 (1.66-9.02) |
| 40-49 | 12/276 |  | 2.37 (1.32-4.25) |
| 50-59 | 18/488 |  | 2.12 (1.32-3.41) |
| 60+ | 16/805 |  | 1.30 (0.79-2.14) |
| Any age | 52/1665 |  | 1.88 (1.42-2.48) |

Due to data availability, this analysis was performed only on the Swedish data

Supplementary Table 2: Breast cancer risk in women with neurofibromatosis type 1 (NF1) – country specific analysis considering also breast cancer diagnosis occurring before the index date

| **Age** | **N breast cancer cases**  **NF1/no NF1** |  | **HR (95% CI)** |
| --- | --- | --- | --- |
| 20-39 | 17/195 |  | 3.08 (1.84-5.14) |
| 30-39 | 17/<172* |  | 3.59 (2.14-6.03) |
| 40-49 | 43/591 |  | 2.80 (1.48-5.30) |
| 50-59 | 50/852 |  | 2.12 (1.59-2.84) |
| 60+ | 38/1313 |  | 1.20 (0.86-1.67) |
| Any age | 148/2951 |  | 1.92 (1.62-2.29) |
| **Sweden** | | | |
| 20-39 | 10/173 |  | 3.05 (1.61-5.78) |
| 30-39 | 10/150 |  | 3.55 (1.87-6.75) |
| 40-49 | 20/527 |  | 2.04 (1.31-3.20) |
| 50-59 | 29/723 |  | 2.30 (1.58-3.33) |
| 60+ | 22/1059 |  | 1.34 (0.87-2.04) |
| Any age | 81/2482 |  | 1.92 (1.54-2.40) |
| **Denmark** | | | |
| 20-39 | 7/22 |  | 3.13 (1.32−7.42) |
| 30-39 | 7/<22* |  | 3.67 (1.52-8.88) |
| 40-49 | 23/64 |  | 3.91 (2.40−6.37) |
| 50-59 | 21/129 |  | 1.87 (1.17−2.99) |
| 60+ | 16/254 |  | 1.01 (0.60−1.71) |
| Any age | 67/469 |  | 1.92 (1.47−2.50) |

Inherently adjusted for birth year

*The actual number of cases in this group cannot be reported because of the Statistics Denmark’s restrictions

Supplementary Table 3: Association between neurofibromatosis type 1 (NF1) and breast cancer risk among women born 1950

| **Age** | | **N breast cancer cases**  **NF1/no NF1** | | **HR (95% CI)** |
| --- | --- | --- | --- | --- |
| 20-39 | | 11/109 | | 3.07 (1.60-5.87) |
| 40-49 | | 27/266 | | 3.08 (1.79-5.31) |
| 50-59 | | 18/362 | | 1.54 (0.95-2.50) |
| Any age | | 62/880 | | 2.09 (1.60-2.74) |
| **Sweden** | | | | |
| 20-39 | 5/90 | | 3.03 (1.23-7.50) | |
| 40-49 | 9/220 | | 2.26 (1.16-4.41) | |
| 50-59 | 8/285 | | 1.58 (0.78-3.21) | |
| Any age | 24/695 | | 1.89 (1.26-2.85) | |
| **Denmark** | | | | |
| 20-39 | 6/19 | | 3.11 (1.23-7.92) | |
| 40-49 | 18/46 | | 3.95 (2.26-6.88) | |
| 50-59 | 10/77 | | 1.50 (0.77-2.93) | |
| Any age | 38/185 | | 2.26 (1.58-3.23) | |

This analysis could not be performed for the age groups 30-39 and 60+
